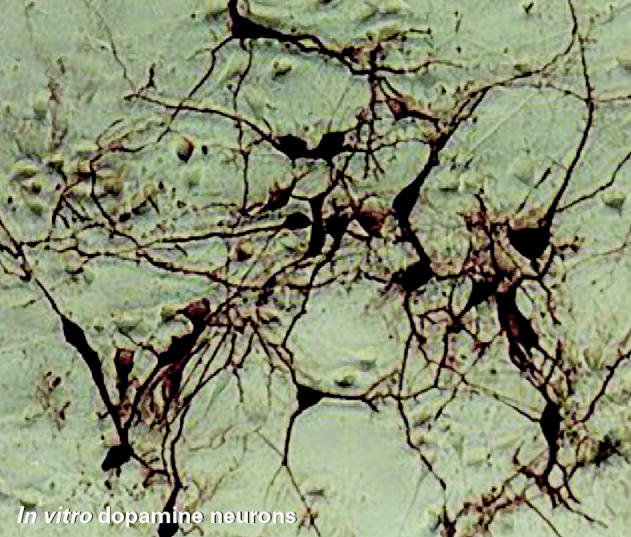# Joining Forces to Understand Parkinson Disease

**Published:** 2005-03

**Authors:** Cindy Lawler

**Affiliations:** lawler@niehs.nih.gov

Parkinson disease (PD) continues to be a high research priority at the NIEHS. The institute currently supports a broad scope of research, from molecular epidemiology studies of environmental risk and protective factors for PD to basic laboratory investigations aimed at developing new animal models, understanding the role of mitochondrial oxidative damage in vulnerable cell populations, and defining transport mechanisms for toxicant entry to the brain. Taken together, results from these studies provide strong support for the idea that typical late-onset PD reflects joint effects of environmental factors and genetic susceptibility.

The NIEHS has special interest in enabling the synthesis of findings emerging across disparate disciplines and research settings in the PD arena. The NIEHS Collaborative Centers for Parkinson’s Disease Environmental Research (CCPDER) Program was initiated in 2002 to strengthen the interchange among geneticists, clinicians, epidemiologists, and scientists engaged in fundamental laboratory research on PD. In June 2004, all NIEHS grantees in the area of neurodegeneration were brought together to share their most recent findings, and to identify and encourage new areas of collaboration among investigators. Most recently, the NIEHS partnered with the National Institute of Neurological Disorders and Stroke to support the development of a database, PD-DOC, that will enable sharing of clinical, pathological, biochemical, and risk factor information among researchers.

A high-priority need that has been identified by PD researchers is for improved methodologies to facilitate data pooling, combined analysis, and comparison of results across epidemiologic studies. In response to this need, the NIEHS has provided support through the CCPDER Program for two meetings that will bring together PD epidemiologists and related experts to develop a series of methodologic recommendations. These will include guidelines for ascertainment and case definition in epidemiologic field studies, and recommendations for both exposure assessment methods and a minimal data set of high-importance exposures to be collected in future investigations. Materials produced through this mechanism will be made widely available through venues such as the CCPDER website, and will be used to guide the development of the epidemiologic component of PD-DOC.

Collectively, these efforts are intended to improve the ability of epidemiologic researchers to identify novel genetic and environmental risk factors for PD, and to inform and be informed by mechanistic laboratory-based research. Ultimately, these data will provide a basis for devising strategies to prevent disease by avoiding or ameliorating harmful exposures and exploiting those exposures that confer protection.

## Figures and Tables

**Figure:**